# The Effectiveness of Multimodality Treatment Including Stabilization Splint and Low‐Level Laser Therapies on Managing Temporomandibular Disorders: A Pilot Randomized Controlled Trial

**DOI:** 10.1002/cre2.70038

**Published:** 2025-01-30

**Authors:** Zahra Afshari, Nazieh Abdollah Kookhi, Mahdi Shamali, Majid Sedaghat Monfared, Sara Tavakolizadeh

**Affiliations:** ^1^ Department of Prosthodontics, School of Dentistry Shahid Beheshti University of Medical Sciences Tehran Iran; ^2^ Department of Prosthodontics, School of Dentistry Kurdistan University of Medical Sciences Sanandaj Iran; ^3^ Research Support Unit, Copenhagen University Hospital – Rigshospitalet Copenhagen Denmark

**Keywords:** low‐level laser therapy, myofascial pain, orofacial pain, stabilization splint, temporomandibular disorders

## Abstract

**Objectives:**

Temporomandibular disorder (TMD) encompasses various clinical issues affecting the temporomandibular joint, masticatory muscles, and surrounding structures. Common symptoms include pain, joint and muscle tenderness, and limited jaw movement. Diverse treatment options have been utilized to manage TMD. However, evidence of the combined modality treatment approach is scarce. This pilot trial aimed to evaluate the effectiveness of combining stabilization splint therapy (SST) with low‐level laser therapy (LLLT) in managing TMD.

**Material and Methods:**

This pilot parallel randomized clinical trial included 30 patients with TMD. Patients were randomly allocated equally into three treatment groups: SST, LLLT, and combined treatment (CT). Outcomes, including pain and functional limitations, were measured at baseline, 1, 2, 3, and 4 weeks after the start of each treatment.

**Results:**

CT was significantly more effective in reducing pain in patients with TMD compared to LLLT and SST. Although patients in the LLLT group reported significantly reduced pain, they still had higher pain scores compared to the SST group at certain time points, indicating that LLLT was not as effective as SST. Most functional limitations were improved over time, more consistently within the CT group, but without significant differences between the three groups. Patients were more satisfied with CT compared to LLLT and SST.

**Conclusions:**

The superior results of the CT group over the SST and LLLT groups were evident in this pilot trial's outcomes. The combined modality treatment approach seems to yield the greatest improvement for TMD patients.

## 1 Introduction

Temporomandibular disorder (TMD) encompasses a range of clinical problems impacting the temporomandibular joint (TMJ), masticatory muscles, and/or surrounding structures (Madani et al. 2020; Shobha et al. 2017). The most frequent manifestation of TMDs is the articular click (Rodrigues et al. 2019). The most common symptoms of TMD include pain, joint and muscle tenderness, and limited jaw movement (Shobha et al. 2017). Nearly two‐thirds of the patients with TMD reported joint pain, one‐third had masticatory muscle pain, and one‐fifth of them experienced limitation of mouth opening (Di Paolo et al. 2013) impacting the ability to eat, speak, sleep (Orzeszek et al. 2023), and perform daily activities (Vrbanović and Alajbeg 2019). A meta‐analysis has reported the overall prevalence of TMD as approximately 31% in the adult population (Valesan et al. 2021).

Because the etiology of TMD is multifactorial, diverse treatment options including pharmacological and nonpharmacological (e.g., behavioral and physical therapies) and invasive (e.g., arthroplasty) therapies have been applied to control pain and recover function of the masticatory system (Madani et al. 2020). There is no specific proven effective therapy that is superior to the other (Vrbanović and Alajbeg 2019). However, the most common approach used for the treatment of TMD is an occlusal splint. Stabilization splints are the most frequently used type in TMD (Vrbanović and Alajbeg 2019). Stabilization splint therapy (SST) by improving occlusal stability and redistributing forces alleviates stress on the TMJ and associated structures and changes neuromuscular activity; it accordingly contributes to relieving symptoms (Vrbanović and Alajbeg 2019). Some studies have demonstrated the clinical efficacy of SST in reducing pain, improving jaw mobility, and decreasing muscle tenderness in TMDs (Vrbanović and Alajbeg 2019; Zhang et al. 2021; Orzeszek et al. 2023). A network meta‐analysis has verified the efficacy of occlusal splint therapy for treating TMDs and suggested that a multimodal approach combining splint therapy with other supplementary treatments could yield the greatest improvement for patients with TMDs (Al‐Moraissi et al. 2020).

Furthermore, low‐level laser therapy (LLLT) has recently received attention due to its conservative nature, easy application, short duration of treatment, and few contraindications (Ahrari et al. 2014; Xu et al. 2018). The underlying mechanism of LLLT therapeutic effects involves the penetration of the laser energy into the tissues, stimulating cellular metabolism, which promotes tissue repair, improves blood circulation, and facilitates nutrient and oxygen delivery to the tissues. Hence, LLLT has anti‐inflammatory properties, reducing pain and swelling in the affected area (Ahrari et al. 2014; Demirkol et al. 2015). Some studies have suggested that LLLT can reduce pain, improve jaw function, and alleviate muscle tenderness in TMD patients (Madani et al. 2020; Rodrigues et al. 2019; Ahrari et al. 2014). A meta‐analysis has confirmed that laser therapy is effective in reducing pain and enhancing mandibular movement in individuals with TMD (Zhang et al. 2023).

The majority of TMD patients respond well to the combination treatment approaches (Vrbanović and Alajbeg 2019). We hypothesize that the combined modality treatment approach, including SST and LLLT, may synergistically enhance treatment outcomes for TMD patients. Therefore, this study aimed to evaluate the effectiveness of combining SST with LLLT for managing TMDs.

## 2 Materials and Methods

### 2.1 Trial Design, Settings, and Participants

This pilot study was a single‐center parallel randomized controlled, open‐label trial conducted from September 2020 to December 2021. Participants were recruited from patients admitted to the Department of Prosthodontics, School of Dentistry, Shahid Beheshti University of Medical Sciences, Tehran, Iran. The inclusion criteria included patients aged 18–60 years with chronic orofacial pain of myogenic origin (≥ 3 months) with/without restriction in opening the mouth who had been diagnosed with TMD based on the DC/TMD diagnostic criteria (Ohrbach and Dworkin 2016; Schiffman et al. 2014) (Axis I; including myalgia, local myalgia, myofascial pain, myofascial pain with referral, and any headache attributed to TMD), had no history of previous treatment for TMD during the last month, and had natural dentition.

The exclusion criteria included recent fracture of mandibular bone, history of recent facial trauma, congenital/developmental disorders of the jaw, systemic musculoskeletal disease, fibromyalgia, previous or current history of cancer, skin lesions at the laser site, pregnancy, diagnosis of TMD of articular origin based on the DC/TMD diagnostic criteria (Schiffman et al. 2014) (including intra‐articular joint and degenerative joint disorders), severe psychological disorders, and complete or partial prosthesis.

### 2.2 Interventions

Patients who met the inclusion criteria were consecutively enrolled in the trial. Patients were randomly allocated to three treatment groups after they had signed an informed consent form.

#### 2.2.1 SST Group

In this group, patients received routine treatment (considered the control group) including SST which was made according to Okeson's instructions (Okeson 2019) (Figure S1). The upper and lower jaw impressions were prepared using premade plastic and condensation silicone impression material (Speedex/putty). The cast of the upper jaw was mounted on a semi‐adjustable nonarcon articulator (MANI Industrial Group, Iran) using a facebow. A Lucia Jig was fabricated using self‐cure resin on the maxillary anterior teeth and was placed in position for 3 min to act as a deprogramming device. Centric relation was recorded using silicone bite registration material (polyvinyl siloxane‐based, extra hard, Kettenbach Futar D bite registration). The mandibular cast was mounted. The stabilization splint was fabricated with hard acrylic resin with a thickness of 2 mm.

The necessary adjustments were made during splint delivery according to the patient's comfort. Patients were examined 3 days after delivery to verify fit, comfort and occlusion, and readjust if it was necessary. The patients were also instructed to use the splint for 12 h a day for 1 month.

#### 2.2.2 LLLT Group

This group underwent LLLT by the first author using a diode laser device (Doctor Smile, Italy) with noncontact mode and continuous wave (Figure S2). We performed LLLT at 0.1 W (100 mW), 810 nm, 6 J/cm^2^, and 60 s per session according to the parameters of LLLT implemented by Shobha et al. (2017). The LLLT was performed in three sessions per week for 3 weeks (a total of nine sessions). The laser is applied to the origin, insertion, and body areas of the masseter muscle and the anterior, middle, and posterior parts of the temporalis muscle, as well as to the center of the upper joint space, about 1 cm in front of the tragus. The patient and clinician both wore protective eyewear during the treatment.

#### 2.2.3 CT Group

In this group, patients received LLLT intervention in addition to the routine treatment. This multimodal treatment included LLLT for 3 weeks (same as the LLLT group), and simultaneously SST for 4 weeks.

### 2.3 Outcome Measures and Data Collection

The outcomes were evaluated at five different time points: before the start of each treatment as baseline (T0), 1 (T1), 2 (T2), 3 (T3), and 4 (T4) weeks after each treatment started. At each assessment time point, the pain intensity, maximum mouth opening (MMO) without assistance, right and left lateral movement of mandible (LMM), and anterior movement of mandible (AMM) were measured.

#### 2.3.1 Pain Intensity

The pain of myogenous TMD was assessed subjectively using a visual analog scale (VAS). This scale consisted of a 10‐cm horizontal line with “0” on the left side indicating no pain and “10” on the right side indicating the most severe pain. The patients were asked to mark the point on the scale that best indicates their perceived pain at rest, during mandibular movement, or palpation of masticatory muscles.

#### 2.3.2 MMO

Unassisted MMO was measured using a manual caliper and recorded in millimeters. Patients were instructed to open their mouths at maximum pain‐free range of motion, then the vertical interincisal distance was measured. Finally, the measured overbite of the patient was added to this recording.

#### 2.3.3 LMM

The right and left LMM were measured using a manual caliper and recorded in millimeters.

First, a vertical line was drawn from the center of one of the central incisor teeth of the upper jaw, which extends to the antagonist incisor tooth in the lower jaw. Then, patients were asked to shift their jaw to one side as far as possible, then the distance between the two lines indicating the lateral movement was measured. This measurement was performed in the right and left lateral movement.

#### 2.3.4 AMM

The AMM, also known as protrusion, was measured using a manual caliper and recorded in millimeters. Patients were asked to move forward the mandible. Then, the horizontal distance between the maxillary and mandibular central incisors was measured, and the amount of overjet of the patient was added to this measurement.

#### 2.3.5 Satisfaction

Patient satisfaction with each treatment was measured after 4 weeks in each group using VAS ranging from 0 (no satisfaction at all) to 10 (definitely satisfied).

### 2.4 Sample Size

The sample size is based on a power analysis for repeated measures ANOVA, comparing three independent groups using the G*Power 3.1.9.2 program. In the program, we set the power at 0.80, a significance level of 0.05, and an effect size of 0.78. The effect size is calculated based on the mean perceived pain intensity of the effect of LLLT (0.82 ± 1.33) and placebo (5.19 ± 2.01) from a previous study (Çetiner, Kahraman, and Yücetas 2006). The sample size was calculated to be nine subjects in each group and considering a 10% drop‐out rate, the total sample size was set at 10 subjects for each group.

### 2.5 Randomization and Allocation

After patients who met the inclusion criteria were consecutively enrolled in the trial, a central randomization unit was contacted by phone to ensure allocation concealment and assign the enrolled patient to each treatment group. The central randomization unit had prepared a list of numbers from 1 to 30 that were allocated randomly to three equal groups using a website (https://www.random.org), which generated a list of random numbers in three columns. Before randomization, the first column was considered the SST group, the second column the LLLT group, and the third column the combined treatment (CT) group.

### 2.6 Ethical Considerations

This trial was performed in accordance with the Declaration of Helsinki (1964) and approved by the Ethics Committee of Shahid Beheshti University of Medical Sciences, Tehran, Iran (No. 59463). The trial was registered at the Iranian Registry of Clinical Trials (www.irct.ir) (No. IRCT20211010052719N1). All participants were informed about the aim and the length of the study, and written informed consent was obtained before inclusion.

### 2.7 Statistical Analysis

All data analyses were conducted in the IBM SPSS Statistics (version 24) with the significance level *p* < 0.05. The chi‐square test and the analysis of variance (ANOVA) were used to compare demographic characteristics among the three groups. Comparison in five time points was conducted for three groups in the form of within‐group and between‐group comparisons using the repeated measures analysis of variance (RMANOVA). Furthermore, the Bonferroni post hoc test was used if the comparison of values in five time points was significant in the RMANOVA within‐group in each group. A regression model was used for post hoc analysis of significant difference scores between the three groups.

## 3 Results

### 3.1 Sample Characteristics

**Figure 1:**
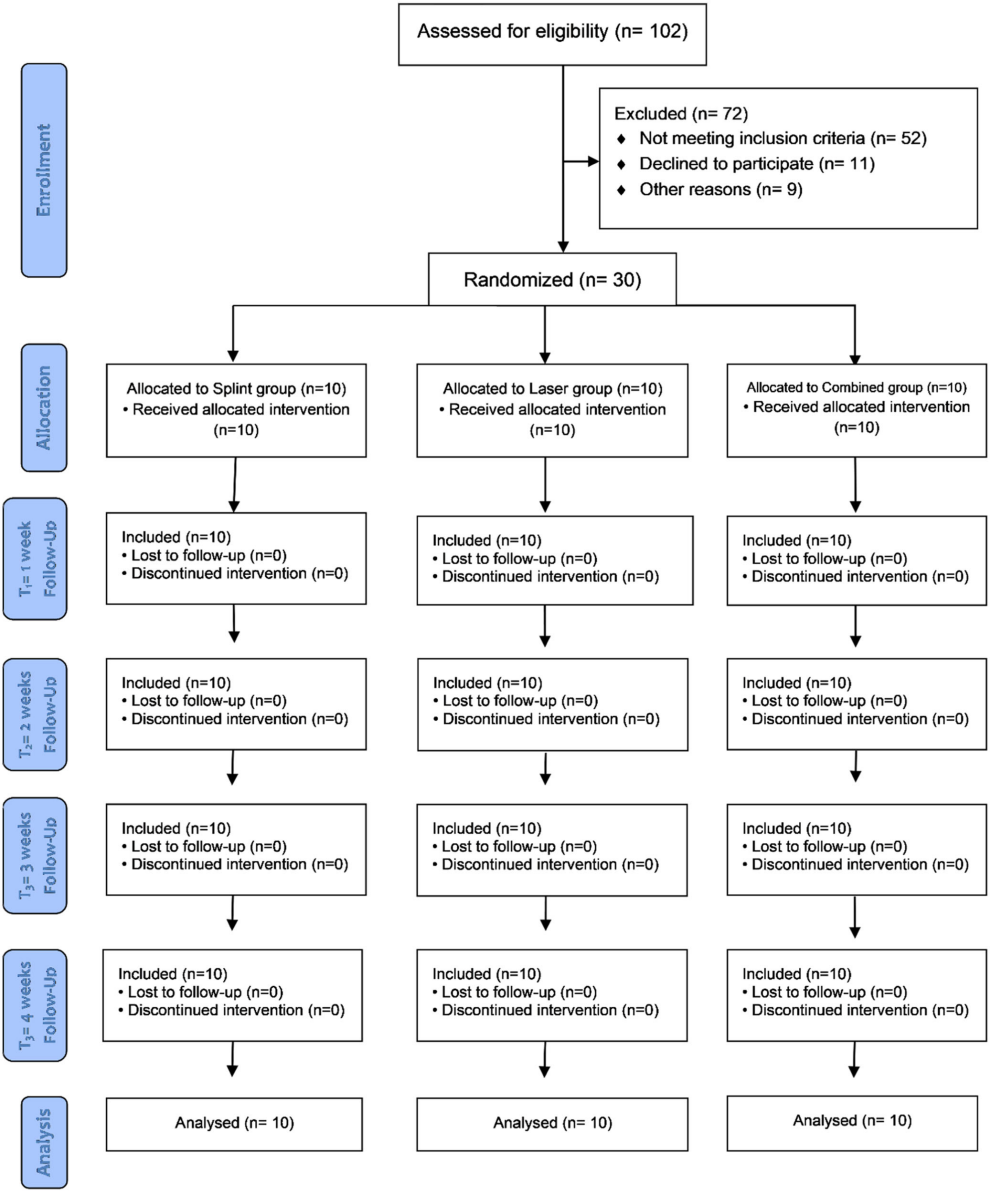
CONSORT flow diagram.

A total of 30 patients were included in this trial. All patients in the three groups completed the trial follow‐ups (Figure 1). Table 1 presents the summary and comparison of patients' characteristics among the three groups. The mean ages of the patients in the CT, SST, and LLLT groups were 39.2, 41.1, and 39.3 years, respectively. Women comprised 70% of the patients in the CT and LLLT groups, and 60% of the patients in the SST group. There were no significant differences in demographic characteristics (*p* > 0.05; Table 1).

**Table 1:** Sample characteristics.

Characteristics	CT	LLLT	SST	*p* value	Test
Sex				0.861^a^	0.300^a^
Male	3 (30%)	3 (30%)	4 (40%)		
Female	7 (70%)	7 (70%)	6 (60%)		
Age (years)	39.2 ± 13.7	39.3 ± 13.4	41.1 ± 12.6	0.937^b^	0.065^b^

*Note:* Data are presented as *n* (%) or mean ± SD.

Abbreviations: CT, combined treatment; LLLT, low‐level laser therapy; SST, stabilization splint therapy.

^a^ The results of the chi‐square test.

^b^ The results of one‐way ANOVA.

### 3.2 Effects on Pain

Within group, RMANOVA showed a significant change in the trend of pain scores from T0 to T4 in the three groups (*p* < 0.001; Table 2). Further analysis with the post hoc test indicated that pain scores during time points T1–T4 were significantly reduced compared to time point T0 in the three groups (Table S1).

**Table 2:** Variable comparison among three groups across five time points.

Variable	Time point	CT	LLLT	SST	RMANOVA^b^
Pain	Baseline	7.5 ± 1	7.7 ± 0.9	7.9 ± 0.7	*F* = *51.6* ***p*** < ***0.001***
	1 week	5.8 ± 0.9	6.9 ± 0.7	6.7 ± 0.7	
	2 weeks	4.3 ± 0.5	6.5 ± 0.5	5.3 ± 0.7	
	3 weeks	3.2 ± 0.8	6.9 ± 0.5	4.6 ± 0.5	
	4 weeks	2 ± 0.5	6.4 ± 0.5	2.9 ± 0.7	
	RMANOVA^a^	*F* = *120.1* ***p*** < ***0.001***	*F* = *7.6* ***p*** < ***0.001***	*F* = *187.2* ***p*** < ***0.001***	
Maximum mouth opening	Baseline	43.4 ± 4.7	44.5 ± 5.8	45.4 ± 6.7	*F* = *0.288* *P* = *0.752*
	1 week	43.8 ± 5	44.6 ± 5.7	45.6 ± 5	
	2 weeks	44.1 ± 5.2	44.9 ± 5.4	45.7 ± 5	
	3 weeks	44.5 ± 5.3	45 ± 5.2	46.1 ± 5	
	4 weeks	44.7 ± 5.2	45.3 ± 5.3	46.6 ± 4.6	
	RMANOVA^a^	*F* = *6.97* ***P*** = ***0.009***	*F* = *5.9* ***P*** = ***0.014***	*F* = *1.74* *P* = *0.216*	
Right lateral movement of mandible	Baseline	8.7 ± 2.4	10.7 ± 2.4	10.1 ± 3.6	*F* = *1.043* *p* = *0.366*
	1 week	9.1 ± 2.3	10.7 ± 2.4	10.5 ± 3.1	
	2 weeks	9.4 ± 1.7	10.8 ± 2.4	10.7 ± 3	
	3 weeks	9.6 ± 1.6	10.9 ± 2.3	10.8 ± 3	
	4 weeks	9.8 ± 1.3	11.1 ± 2.1	10.8 ± 3.1	
	RMANOVA^a^	*F* = *4.64* ***p*** = ***0.038***	*F* = *3.5* ***p*** = ***0.016***	*F* = *4.8* ***p*** = ***0.003***	
Left lateral movement of mandible	Baseline	8.2 ± 2.4	10.5 ± 1.7	10 ± 3.1	*F* = *2.203* *p* = *0.130*
	1 week	8.6 ± 2.3	10.6 ± 1.6	10.3 ± 3	
	2 weeks	8.7 ± 2	10.6 ± 1.6	10.4 ± 3.1	
	3 weeks	8.8 ± 1.9	10.7 ± 1.7	10.5 ± 2.9	
	4 weeks	9.1 ± 1.7	11 ± 1.4	10.6 ± 2.9	
	RMANOVA^a^	*F* = *3.8* ***p*** = ***0.011***	*F* = *2.32* *p* = *0.075*	*F* = *1.55* *p* = *0.208*	
Anterior movement of mandible	Baseline	6.5 ± 1.7	7.5 ± 1.9	7.8 ± 2.3	*F* = *0.885* *p* = *0.424*
	1 week	6.8 ± 1.7	7.6 ± 1.9	8.1 ± 2.2	
	2 weeks	7 ± 1.9	7.6 ± 1.8	8.2 ± 2.2	
	3 weeks	7.2 ± 2.4	7.7 ± 1.7	8.3 ± 2.3	
	4 weeks	7.3 ± 2.6	7.9 ± 1.6	8.4 ± 2.4	
	RMANOVA^a^	*F* = *2.12* *p* = *0.173*	*F* = *1.93* *p* = *0.175*	*F* = *1.7* *p* = *0.213*	
Overall satisfaction	4 weeks	8.8 ± 0.8	7.3 ± 0.7	7.5 ± 0.8	*F* = *11.06* c ***p*** < ***0.001*** c

*Note:* All data are presented as mean ± SD.

Abbreviations: CT, combined treatment; LLLT, low‐level laser therapy; RMANOVA, repeated measures analysis of variance; SST, stabilization splint therapy.

^a^ RMANOVA of values changes within each group.

^b^ RMANOVA of comparison between the three groups in five time points (Interaction between groups and time).

^c^ Results for one‐way ANOVA comparison among three groups.

**Figure 2:**
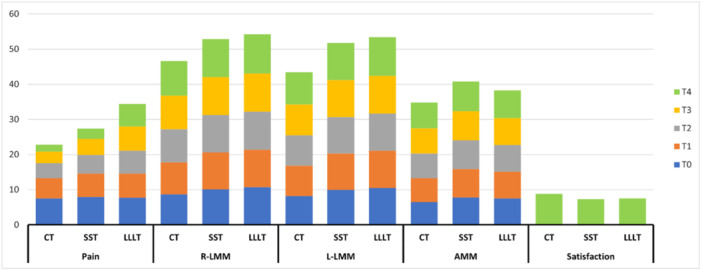
Three‐dimensional stacked column for the outcome's comparison among three groups across five time points. AMM, anterior movement of mandible; CT, combined treatment; SST, stabilization splint therapy; LLLT, low‐level laser therapy; L‐LMM, left lateral movement of mandible; R‐LMM, right lateral movement of mandible; T0, time point baseline; T1, 1 week; T2, 2 weeks; T3, 3 weeks; T4, 4 weeks after each treatment started.

Between groups comparison with RMANOVA of the interaction between pain scores and time (trend of change in pain scores from T0 to T4) was statistically significant (*p* < 0.001; Table 2 and Figure 2), indicating a significant difference in pain scores among the three groups. Further post hoc analysis with a regression model using SST as a reference group showed that patients in the CT group had significantly lower pain scores in all time points from T1 to T4 compared to the SST group. However, patients in the LLLT groups had significantly higher pain scores in the time points T2, T3, and T4 compared to the SST group (Table S2).

### 3.3 Effects on MMO

**Figure 3:**
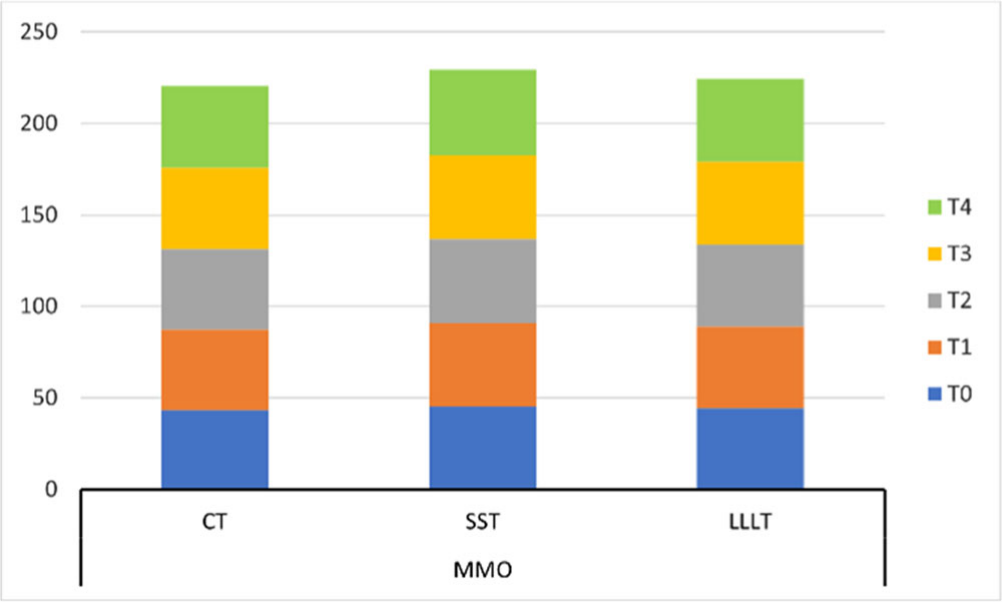
Three‐dimensional stacked column for the comparison of maximum mouth opening among three treatment groups across five time points. CT, combined treatment; LLLT, low‐level laser therapy; MMO, maximum mouth opening; SST, stabilization splint therapy; T0, time point baseline; T1, 1 week; T2, 2 weeks; T3, 3 weeks; T4, 4 weeks after each treatment started.

Within group, RMANOVA showed a significant change in the trend of MMO scores from T0 to T4 in CT and LLLT groups (*p* = 0.009 and *p* = 0.014 respectively; Table 2). Further post hoc analysis indicated that MMO scores of the CT group during time points T1, T3, and T4 were significantly increased compared to time point T0 (Table S1). However, in the LLLT group, MMO scores were significantly increased during time points T2 and T4 compared to time point T0. In the SST group, there was no significant change in the trend of MMO scores from T0 to T4 (*p* = 0.216). Between groups, comparison with RMANOVA showed no significant difference in MMO scores among the three groups across five time points (*p* = 0.752; Table 2 and Figure 3).

### 3.4 Effects on LMM

Regarding right LMM scores, within‐group RMANOVA showed a significant change in the trend of LMM scores from T0 to T4 in the three groups (*p* < 0.05; Table 2). Further post hoc analysis indicated that right LMM scores were significantly increased during time points T1–T4 in the CT group, time point T4 in the LLLT group, and time points T2–T4 in the SST group compared to time point T0 (Table S1).

Regarding left LMM scores, within‐group RMANOVA showed a significant change in the trend of LMM scores from T0 to T4 only in the CT group (*p* = 0.011; Table 2). Further post hoc analysis indicated that right LMM scores in the CT group were significantly increased during time points T1 and T4 compared to time point T0 (Table S1). Between groups, comparison with RMANOVA showed no significant difference in right and left LMM scores among the three groups across five time points (*p* = 0.366 and *p* = 0.130, respectively; Table 2 and Figure 2).

### 3.5 Effects on AMM

Within group, RMANOVA showed no significant change in the trend of AMM scores from T0 to T4 in the three groups (*p* > 0.05; Table 2). Between groups comparison with RMANOVA showed no significant difference in AMM scores among the three groups across five time points (*p* = 0.424; Table 2 and Figure 2).

### 3.6 Effects on Satisfaction

Patients in the CT group had significantly higher satisfaction with the treatment compared to the LLLT and SST groups (8.8 vs. 7.3 and 7.5, respectively; *p* < 0.001; Table 2 and Figure 2).

## 4 Discussion

The key findings of this trial indicate that CT is significantly more effective in reducing pain in patients with TMD compared to LLLT and SST. Although LLLT reduced pain, it showed higher pain scores compared to SST at certain time points indicating that it was not as effective as SST. MMO improved over time within CT and LLLT groups but without significant differences between the three groups. Right LMM improved over time within all groups, more consistently in the CT group, but without significant differences between the three groups. Left LMM improved over time within only the CT group, but without significant differences between the three groups. AMM had no significant changes over time within any group and between groups. The overall satisfaction with the treatment was significantly higher among the CT group.

Our findings align with some studies but contrast with others. For instance, one study (Öz et al. 2010) compared LLLT (twice a week for a total of 18 sessions) with SST (3 months) and reported no significant difference in the amount of patients' pain reduction between the two groups; each treatment method was equally effective in reducing pain. Another study (Demirkol et al. 2015) compared LLLT (10 days) and SST (3 weeks) with a placebo group and reported that both LLLT and SST groups showed a significant reduction in pain after the end of the treatment compared to the placebo group, and they showed that LLLT was as effective as SST in reducing pain of the patients with TMD. In contrast, one study (Maracci et al. 2022) compared LLLT (4 days) and occlusal splint (1 month) with a placebo group after 1 month of each treatment and reported that the occlusal splint group showed a significant reduction in myofascial pain after the end of the treatment, whereas LLLT and placebo groups did not. Although the effect of LLLT on TMD has been investigated in many studies, there is still no consensus on a precise standard laser therapy protocol and parameters such as laser application period, laser dosage, session interval, or total duration of treatment with laser (Demirkol et al. 2015). Nagata et al. (2015) showed that SST alone or in combination with other treatment methods was not effective in increasing the MMO after each treatment. In contrast, Vrbanović and Alajbeg (2019) showed a significant improvement in MMO with SST after 6 months of follow‐up. Moreover, some studies reported a positive effect of LLLT in improving MMO, LMM, and AMM (Madani et al. 2020).

LLLT induces therapeutic effects through a photochemical mechanism similar to photosynthesis, necessitating adequate photon delivery to the target tissues (Ren et al. 2022). The efficacy of LLLT depends on several factors, including the laser's wavelength, power output, and the tissue's absorption characteristics and penetration depth (Xu et al. 2018). Infrared light, such as the 810 nm wavelength used in our study, penetrates deeper into tissues compared to red light (wavelengths below 700 nm), resulting in more pronounced analgesic effects. Previous research supports the superior tissue penetration and analgesic efficacy of infrared light (e.g., 904 nm) compared to red light (e.g., 632.8 nm) (Ren et al. 2022; Chang et al. 2014). However, despite using an optimal wavelength in our study, LLLT alone did not achieve the same level of pain reduction as CT and SST, suggesting the need for further optimization of treatment parameters and potentially combining different wavelengths for enhanced therapeutic effects. The superior efficacy of CT highlights the potential benefits of a CT approach. Future studies should focus on optimizing LLLT parameters, exploring the synergistic effects of combining different wavelengths, and integrating LLLT with other therapeutic modalities to maximize patient outcomes. Additionally, larger scale studies with diverse patient populations and longer follow‐up periods are needed to validate these findings and determine the long‐term benefits of these treatments. Exploring the combination of CT and LLLT with other therapeutic modalities could also provide insights into more comprehensive management strategies for TMD.

Our study has several limitations that should be acknowledged. First, the sample size was relatively small, and we did not include a placebo group, which may limit the generalizability of our findings. Larger, multicenter trials are needed to confirm the results. Second, the follow‐up period was relatively short, restricting our ability to assess the long‐term efficacy and durability of the treatments. Long‐term studies are essential to understand the lasting impacts of LLLT and CT on TMD. Lastly, the study did not explore the psychosocial aspects of TMD, which are critical in understanding the full spectrum of the disorder and its management. Incorporating psychosocial evaluations and interventions in future research could provide a more comprehensive understanding of TMD management.

## 5 Conclusions

By considering the limitation of this pilot study, our results demonstrated that patients in the CT group had significantly lower pain and higher satisfaction compared to both LLLT and SST groups. This finding aligns with previous research indicating that combining different therapeutic modalities enhances treatment outcomes in TMD management. Future studies should continue to refine and optimize LLLT protocols, explore additional combinations of therapeutic modalities within CT, and further investigate long‐term outcomes to guide clinical practice effectively.

## Author Contributions

Zahra Afshari, Nazieh Abdollah Kookhi, Mahdi Shamali, Majid Sedaghat Monfared, and Sara Tavakolizadeh contributed to the study conception and design. Material preparation and data collection were performed by Zahra Afshari. Data analyses were performed by Mahdi Shamali and Zahra Afshari. The first draft of the manuscript was written by Zahra Afshari. Zahra Afshari, Nazieh Abdollah Kookhi, Mahdi Shamali, Majid Sedaghat Monfared, and Sara Tavakolizadeh critically revised the manuscript. All authors read and approved the final manuscript.

## Conflicts of Interest

The authors declare no conflicts of interest.

## Supporting information

Supporting information.

Supporting information.

## Data Availability

The data that support the findings of this study are available from the corresponding author upon reasonable request.
