# Supplementary material for: The Effectiveness of Multimodality Treatment Including Stabilization Splint and Low‐Level Laser Therapies on Managing Temporomandibular Disorders: A Pilot Randomized Controlled Trial
Source: Clin Exp Dent Res. 2025 Jan 30;11(1):e70038. doi: 10.1002/cre2.70038 (PMC11780597; doi:10.1002/cre2.70038)
Supplement: Supplementary file 1 — Supporting information. [file CRE2-11-e70038-s001.docx]

**Supp. Table 1.** Post hoc pairwise within group comparisons of variables over time adjusted by Bonferroni.

| Variable | Treatment group | Time point (i) | Time point (j) | Mean difference  (i-j) | SE | P value |
| --- | --- | --- | --- | --- | --- | --- |
| Pain | CT | T0 | T1 | 1.700 | 0.213 | **<0.001** |
|  |  |  | T2 | 3.200 | 0.327 | **<0.001** |
|  |  |  | T3 | 4.300 | 0.367 | **<0.001** |
|  |  |  | T4 | 5.500 | 0.342 | **<0.001** |
|  | LLLT | T0 | T1 | 0.800 | 0.133 | **<0.001** |
|  |  |  | T2 | 1.200 | 0.200 | **<0.001** |
|  |  |  | T3 | 0.800 | 0.291 | **0.022** |
|  |  |  | T4 | 1.300 | 0.396 | **0.009** |
|  | SST | T0 | T1 | 1.200 | 0.291 | **0.003** |
|  |  |  | T2 | 2.600 | 0.221 | **<0.001** |
|  |  |  | T3 | 3.300 | 0.153 | **<0.001** |
|  |  |  | T4 | 5.000 | 0.211 | **<0.001** |
| Maximum mouth opening | CT | T0 | T1 | –0.400 | 0.163 | **0.037** |
|  |  |  | T2 | –0.700 | 0.335 | 0.066 |
|  |  |  | T3 | –1.10 | 0.375 | **0.017** |
|  |  |  | T4 | –1.30 | 0.423 | **0.013** |
|  | LLLT | T0 | T1 | –0.100 | 0.100 | 0.343 |
|  |  |  | T2 | –0.400 | 0.163 | **0.037** |
|  |  |  | T3 | –0.500 | 0.224 | 0.052 |
|  |  |  | T4 | –0.800 | 0.291 | **0.022** |
| Right lateral movement of mandible | CT | T0 | T1 | -0.400 | 0.163 | **0.037** |
|  |  |  | T2 | -0.700 | 0.260 | **0.025** |
|  |  |  | T3 | -0.900 | 0.348 | **0.029** |
|  |  |  | T4 | -1.100 | 0.433 | **0.032** |
|  | LLLT | T0 | T1 | .000 | .000 | 1.000 |
|  |  |  | T2 | -0.100 | 0.100 | 0.343 |
|  |  |  | T3 | -0.200 | 0.133 | 0.168 |
|  |  |  | T4 | -0.400 | 0.163 | **0.037** |
|  | SST | T0 | T1 | -0.400 | 0.221 | 0.104 |
|  |  |  | T2 | -0.600 | 0.221 | **0.024** |
|  |  |  | T3 | -0.700 | 0.260 | **0.025** |
|  |  |  | T4 | -0.700 | 0.260 | **0.025** |
| Left lateral movement of mandible | CT | T0 | T1 | -0.400 | 0.163 | **0.037** |
|  |  |  | T2 | -0.500 | 0.269 | 0.096 |
|  |  |  | T3 | -0.600 | 0.267 | 0.051 |
|  |  |  | T4 | -0.900 | 0.348 | **0.029** |

CT: combined treatment; LLLT: low-level laser therapy; SST: stabilization splint treatment; T0: Baseline; T1: 1 week; T2: 2 weeks; T3: 3 weeks; T4: 4 weeks; SE; standard error.

**Supp. Table 2.** Post hoc between groups comparisons of pain scores in each time point.

| **Time point** | **Group** | **β** | **SE** | **t** | **P value** | **95% CI** | |
| --- | --- | --- | --- | --- | --- | --- | --- |
| **Baseline** | CT | -0.400 | 0.417 | -0.959 | 0.346 | -1.256 | 0.456 |
|  | LLLT | -0.200 | 0.417 | -0.479 | 0.636 | -1.056 | 0.656 |
|  | SST–reference­ | # |  |  |  |  |  |
| **1 week** | CT | -0.900 | 0.351 | -2.567 | **0.016** | -1.619 | -0.181 |
|  | LLLT | 0.200 | 0.351 | 0.570 | 0.573 | -0.519 | 0.919 |
|  | SST–reference­ | # |  |  |  |  |  |
| **2 weeks** | CT | -1.000 | 0.254 | -3.939 | **<0.001** | -1.521 | -0.479 |
|  | LLLT | 1.200 | 0.254 | 4.727 | **<0.001** | 0.679 | 1.721 |
|  | SST–reference­ | # |  |  |  |  |  |
| **3 weeks** | CT | -1.400 | 0.284 | -4.927 | **<0.001** | -1.983 | -0.817 |
|  | LLLT | 2.300 | 0.284 | 8.094 | **<0.001** | 1.717 | 2.883 |
|  | SST–reference­ | # |  |  |  |  |  |
| **4 weeks** | CT | -0.900 | 0.262 | -3.429 | **0.002** | -1.439 | -0.361 |
|  | LLLT | 3.500 | 0.262 | 13.335 | **<0.001** | 2.961 | 4.039 |
|  | SST–reference­ | # |  |  |  |  |  |

β: unstandardized regression coefficients; SE: standard error; CI: confidence interval; CT: combined treatment; LLLT: low-level laser therapy; SST: stabilization splint treatment.

# This parameter is set to zero.
