# Supplementary material for: The Effectiveness of Multimodality Treatment Including Stabilization Splint and Low‐Level Laser Therapies on Managing Temporomandibular Disorders: A Pilot Randomized Controlled Trial
Source: Clin Exp Dent Res. 2025 Jan 30;11(1):e70038. doi: 10.1002/cre2.70038 (PMC11780597; doi:10.1002/cre2.70038)

Supplementary Figure 1. Fabricating a stabilization splint.

(A) Record of centric relation; (B) and (C) Articulator mounting and wax-up; (D) stabilization splint

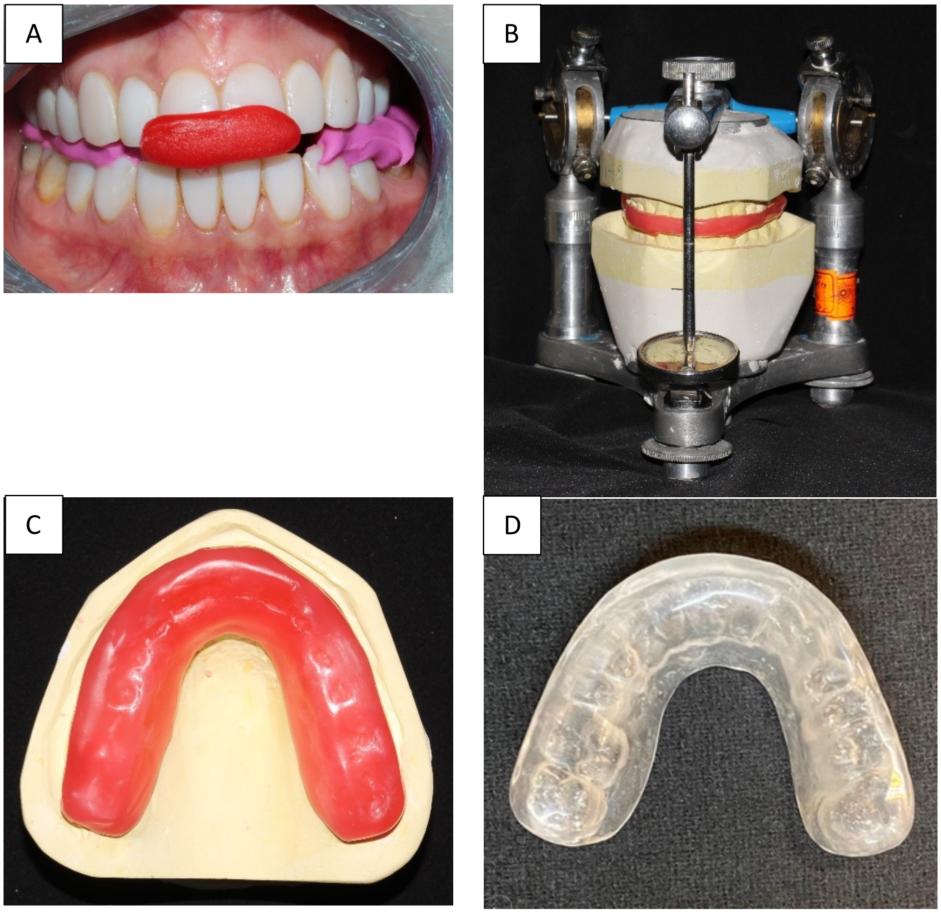

Supplementary Figure 2. The diode laser device

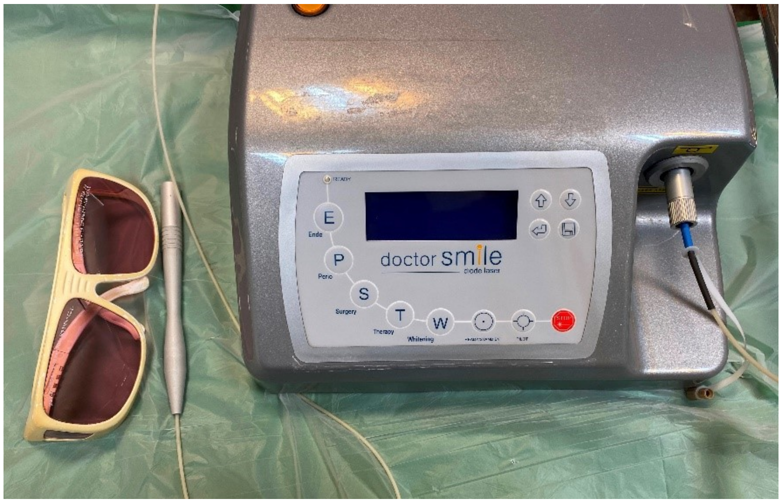

Supplement: Supplementary file 2 — Supporting information. [file CRE2-11-e70038-s002.pdf]
